# Exercise rescues mitochondrial coupling in aged skeletal muscle: a comparison of different modalities in preventing sarcopenia

**DOI:** 10.1186/s12967-021-02737-1

**Published:** 2021-02-16

**Authors:** Colin Harper, Venkatesh Gopalan, Jorming Goh

**Affiliations:** 1 Clinical Translation Unit (CTU), Tulane University, New Orleans, USA; 2 Agency for Science, Technology & Research (A*STAR), Singapore Bioimaging Consortium (SBIC), Singapore, Singapore; 3 Healthy Longevity Translational Research Program, Yong Loo Lin School of Medicine, National University of Singapore (NUS), Singapore, Singapore; 4 Department of Physiology, Yong Loo Lin School of Medicine, National University of Singapore, Singapore, Singapore; 5 Centre for Healthy Longevity, National University Health System (NUHS), Singapore, Singapore

**Keywords:** Skeletal muscle, Mitochondria, Aging, Exercise

## Abstract

Skeletal muscle aging is associated with a decline in motor function and loss of muscle mass- a condition known as sarcopenia. The underlying mechanisms that drive this pathology are associated with a failure in energy generation in skeletal muscle, either from age-related decline in mitochondrial function, or from disuse. To an extent, lifelong exercise is efficacious in preserving the energetic properties of skeletal muscle and thus may delay the onset of sarcopenia. This review discusses the cellular and molecular changes in skeletal muscle mitochondria during the aging process and how different exercise modalities work to reverse these changes. A key factor that will be described is the efficiency of mitochondrial coupling—ATP production relative to O_2_ uptake in myocytes and how that efficiency is a main driver for age-associated decline in skeletal muscle function. With that, we postulate the most effective exercise modality and protocol for reversing the molecular hallmarks of skeletal muscle aging and staving off sarcopenia. Two other concepts pertinent to mitochondrial efficiency in exercise-trained skeletal muscle will be integrated in this review, including- mitophagy, the removal of dysfunctional mitochondrial via autophagy, as well as the implications of muscle fiber type changes with sarcopenia on mitochondrial function.

## Introduction

Harman’s free radical theory of aging proposed that aging is associated with the accumulation of oxidative damage to proteins, lipids and DNA in living tissues [1]. Such damages could impair the normal functioning of the adenosine triphosphate (ATP) producing organelles—the mitochondria, resulting in deficient energy production. Studies in older humans have documented an age-related decrease in aerobic capacity, suggesting that mitochondrial dysfunction or loss in skeletal muscle could be the underlying mechanism. Indeed, a number of hallmark changes occur in skeletal muscle mitochondria with aging including increases in mitochondrial DNA (mtDNA) deletions [2], reductions in enzyme activity and mtDNA content, and increases in oxidative stress [3]. Some studies, however, implicate increased sedentarism which usually accompanies human aging as a greater culprit for such decline of skeletal muscle function than the aging itself.

The first part of this review will briefly describe the mechanisms involved in oxidative phosphorylation in the mitochondria of skeletal muscle. Subsequently, there will be a discussion on the hallmark mitochondrial changes that occur in skeletal muscle with aging as well as an examination of the evidence for and against aging, or physical inactivity, being the primary cause of abnormal energetics in aged skeletal muscle. Regardless of whether sedentary living or aging per se is at the root of declining energy metabolism, physical demands of daily living present a challenge to older individuals. Hence, the final part of this review will investigate the mechanisms by which different exercise forms restore mitochondrial energetics and compare these different exercise forms in their effectiveness to prevent mitochondrial aging and intercept sarcopenic development in older adults.

## Skeletal muscle mitochondria—powerhouses to drive locomotion

Energy is needed for movement. In human locomotion, this energy is derived from ATP, a large percentage of which is generated in the mitochondria, via the process of oxidative phosphorylation. Two membranes envelop the mitochondrion—the inner membrane and the outer membrane, where the former allows the passage of H^+^, which forms the crux of the mechanism for energy transduction during oxidative phosphorylation [4]. Small molecules and ions pass through transmembrane channels embedded in the outer membrane.

Cristae are the invaginations of the inner membrane, and the matrix, the compartment lying within the inner membrane, is the site in which the reducing equivalents nicotinamide adenine dinucleotide (NADH) and flavin adenine dinucleotide (FADH_2_) transfer electrons to the electron transport chain (ETC) [5]. Preceding events at the ETC is the oxidation of substrates through the Krebs cycle, which generates the bulk of the reducing equivalents described above. The ETC is a system of four enzyme complexes [5] that perform sequential reduction–oxidation reactions (4), systematically transporting the electrons across each of the complexes to ultimately reduce O_2_ and generate H_2_O, while pumping protons out of the matrix along the way.

As electrons are moved from complex I to complex IV, protons are actively translocated by complexes I, III and IV to the intermembrane space of the mitochondrion. This movement of ions generates an electrochemical gradient between the intermembrane space and the matrix [5], with the matrix becoming more negatively charged and the intermembrane space becoming more positively charged [4]. Consequently, as protons flow down the electrochemical gradient and through the ATP synthase (complex V) into the matrix, adenosine diphosphate (ADP) is phosphorylated to ATP [4].

The capacity for, and efficiency of, ATP synthesis are two concepts that will be relevant for the discussion on mitochondrial function in this manuscript. How much ATP is synthesized depends on the flux through the ETC as well as the rate of oxygen uptake [5]. These factors are, in turn, determined by the energy demand of the cell, which, when high, ATP breakdown is augmented. This then increases [ADP] and [P_i_], which stimulate ATP regeneration [4]. Therefore, energy demand drives ATP synthesis. An important concept in understanding mitochondrial oxidative phosphorylation is the distinction between State 3 and State 4 respiration. These two terms define the metabolic state of mitochondria; state 3 respiration takes place when there are both ADP and other respiratory substrates available, whereas State 4 respiration occurs in the absence of ADP [4]. State 3 respiration, also called “active respiration”, is characterized by maximal ATP synthesis and oxygen uptake, during which the rate of respiration is 5–8 times faster than that of State 4 [4]. It has been described that 60–70% of mammalian aerobic respiration occurs in State 4, with the rest occurring in State 3.

Whether aging itself or sedentary living typically associated with old age is the phenomenon primarily responsible for declining ATP synthesis, is a subject of continued debate. The coupling of ATP synthesis per molecule of oxygen consumed is known as the P/O ratio [6]. In essence, the P/O ratio defines the coupling efficiency of mitochondrial oxidative phosphorylation and is a useful working tool to quantitatively compare rates of ATP production relative to oxygen consumption. Energy production and efficiency relevant to the discussion of aging skeletal muscle will be the basis of discussion in the next section.

## Does age-associated oxidative stress cause mitochondrial impairment in skeletal muscle?

Reactive oxygen species (ROS) are generated during normal respiration, with approximately 0.1–2% of O_2_ reduced to form superoxide [5, 7], the main source of oxidative stress in the cell [8], which is generated primarily at complexes I and III of the ETC [9]. Most of the superoxide produced in the mitochondria is released to the matrix, with a smaller proportion channeled to the intermembrane space [4]. Other ROS generated in the mitochondria include H_2_O_2_ and NO which, together with superoxide, augment oxidation of lipids, proteins and DNA of mitochondria [5], leading to damage of these constituents. Higher rates of production of ROS such as H_2_O_2_ occur during State 4 respiration than during State 3 respiration [4]. It was also reported that State 4 respiration was higher in skeletal muscle of 24-month old rats, compared with 4-month old rats, while the ATP produced per O_2_ consumed (P/O ratio) was lower in aged rats relative to young rats [10]. These results have implications that give credence to the oxidative damage theory of aging. That is, mitochondria of older organisms are less efficient at generating ATP for a given amount of oxygen, possibly as a consequence of increased ROS damage to mitochondria, which is also associated with increased H^+^ leak and increased uncoupling. This mechanism could explain the age-related decline in muscle function.

The studies above summarize the mitochondrial theory of aging, an extension of the free radical theory of aging, which attributes free radical damage on mitochondrial components as the cause of mitochondrial dysfunction [4]. Because the respiratory chain is a major producer of ROS, the membrane’s proximity to the mitochondrial genome, along with mtDNA’s lack of protective histones and less robust repair mechanisms compared to the nuclear genome [9], makes mtDNA especially susceptible to ROS-induced mutations. In a nutshell, the mitochondrial theory of aging states that accumulation of mtDNA mutations results in aberrant or decreased expression of ETC protein complexes, which in turn leads to progressive uncoupling of the ETC and thereby increased generation of radical species, giving rise to a vicious cycle of further oxidative damage, mtDNA mutations, and respiratory uncoupling. Supporting the idea that free radical-induced damage is correlated with aging, a study by Mansouri and colleagues [8] demonstrated that mitochondria isolated from the hind-limb skeletal muscle of old (28–29 month) mice produced 50–80% higher H_2_O_2_, depending on substrate added, than young (6–8 month) mice. The older mice also had higher oxidative damage to mtDNA, as evidenced by higher levels of 8-oxo-2deoxyguanosine (oxo8dG). Congruently, age-related functional deficits were reflected by a 30% reduction in ATP production in the old mice, compared with the young mice.

However, while high levels of ROS damage macromolecular structures, low levels of ROS are thought to confer greater stress resistance and even lifespan extension through an adaptive defense response known as “mitohormesis” [11]. Specifically, low level ROS presence is thought to induce mitochondrial stress response mechanisms that enable better tolerance of subsequent stressors as well as induce mtDNA repair mechanisms. Therefore, there seems to be an adaptively beneficial range of ROS presence beyond which the effects grow increasingly deleterious on mtDNA. With aging and sedentarism, however, the upper threshold of this range is far surpassed. Thus, exercise that increases coupling efficiency, and thereby mitigates electron leakage and excessive ROS generation, could restore the range to a baseline, favorable levels as seen in healthy adults.

More importantly, duration (acute vs chronic) seems to be a major determinant of whether ROS production is adaptive or destructive. Acute, exercise-induced ROS generation is associated with the benefits of increased mitochondrial biogenesis and muscle hypertrophy, while chronic basal elevations of ROS generally act pathologically [12]. For example, it has recently been shown that aerobic exercise induces acute bouts of oxidative stress which trigger mitohormesis via activation of the redox-sensitive transcription factor Nrf2, an endogenous antioxidant gene, resulting in protection against sarcopenia [13].

## Mitochondrial DNA deletion-mutations in aging muscle

The aforementioned findings by Mansouri et al. are consistent with other animal and human studies that reported the following age-related changes in skeletal muscle mitochondria: increased mtDNA deletions-mutations [14–18] and damage [14], decreased mitochondrial DNA abundance [14, 19] and decreased mitochondrial protein synthesis [20]. In fact, the prevalence of mtDNA deletions in human skeletal muscle increases so predictably and proportionately with age that it has been considered for use in age estimation [21].

Increased mtDNA deletion in biopsies obtained from the vastus lateralis of older human subjects was detected with polymerase chain reaction (PCR) [16] and found to be correlated with a cytochrome-c oxidase negative/succinate dehydrogenase-hyperreactive (COX^−^/SDH^++^) muscle phenotype. This peculiar phenotypic expression is typical of impaired oxidative phosphorylation, where the morphological change in the muscle manifests as a "ragged red" appearance, owing to the staining pattern used with the modified Gomori technique [17]. Mechanistically, this indicates SDH activity accompanied by decreased cytochrome-c oxidase (COX) activity, and thus is a manifestation of aberrant ETC activity. The researchers of this study examined the abundance of mtDNA deletion-mutation along the length of the muscle fibers and demonstrated that the distribution of the deletion-mutations clustered at regions where there was expression of the COX^−^/SDH^++^ phenotype. Their results supported the hypothesis that mitochondrial abnormalities are concomitant with aging. The evidence, however, is not convincing because muscle samples were obtained autopsies of a small sample of subjects (*N* = 12) that varied in age, causes of death and body weight. This makes the older subjects in the sample heterogeneous and unlikely to be representative of people in their age group.

Two previous studies by the same group [15, 17] assessed mitochondrial function of different muscle groups isolated from rats. In those studies, age correlated with the expression of the COX^−^/SDH^++^ phenotype. They also reported that the cross-sectional area of the fibers decreased within the COX^−^/SDH^++^ region and that there was fiber splitting in those regions. Interestingly, the length of the abnormal region was associated with fiber atrophy: the mean length of the COX^−^/SDH^++^ region was 239 ± 17 microns for 5-month-old rats, 266 ± 33 microns for 18-month-old rats, and 267 ± 14 microns for 38-month-old rats [15]. Hence, systematic atrophy in aged skeletal muscle fibers of rats appeared to be associated with abnormal ETC phenotypic expression.

In an effort to elucidate a more specific role of mtDNA deletion-mutations in muscle aging, Herbst and colleagues [18] quantified the amount of mutant and wild-type mitochondrial genomes along the lengths of muscle fibers dissected from aged (36-month) rats. They reported that within each COX^−^/SDH^++^ region, the abundance of mutated mitochondrial genomes was greater than 90%. Wild-type genomes, on the other hand, were localized at areas that were further from regions with the aberrant expression. Therefore, it is possible that the genetic machinery is mutated to such a detrimental extent that the normal phenotypic expression of the muscle fibers is impaired. To further confirm the causality between mtDNA deletions and sarcopenia, Herbst et al. [22] pharmacologically induced the same type of mtDNA deletion mutation that is observed to accumulate in aged skeletal muscle in aged rat muscle. Both muscle fiber number and muscle mass decreased in proportion with the amount of deletion induced, affirming the fact that ETC-altering deletion-mutations of mtDNA contribute to skeletal muscle deterioration with aging.

Subsequently, Herbst et al. [23] speculated that maladaptive mitochondrial biogenesis may act as the driving force behind the amplification and spread of mtDNA deletion-mutations, finding that induction of mitochondrial biogenesis via treatment of middle-aged rats with β-Guanidinopropionic acid resulted in a 3.7-fold increase in the number of ETC-aberrant fibers. More recently, the mechanism for propagation of ETC-aberrant fibers has been further elucidated by analysis of isolated human skeletal muscle tissue [24]. It was found that ETC-abnormal fibers containing mtDNA mutations were localized with elevations of mitochondrial mass and mtDNA copy number. It is speculated, then, that the energetic insufficiencies caused by mtDNA deletion-mutations and the accompanying ETC defects induce mitochondrial biogenesis in a compensatory cellular effort to restore adequate energy production, and thereby engender a vicious cycle of propagating these mutations [23]. Specifically, it is believed, mitochondrial biogenesis first generates focal accumulation of clonal deletion-mutated mtDNA, and that mutant mitochondria subsequently propagate transversely along the muscle fiber following mitochondrial networks, hence the anisotropic mosaic pattern of COX-/SHD^++^ fibers [24].

In any case, mtDNA mutations are relevant to the conversation of mitochondrial energetic decline as their rates have been correlated with bioenergetic deficiency, particularly of complex IV, and muscle atrophy [25]. Complex IV, being the terminal electron acceptor of the ETC, plays a crucial role here in that increasing its activity may mitigate electron leakage and thereby reduce ROS production [26]. Given that ET [14, 27, 28] and RT [2] have been shown to upregulate complex IV activity, dampening mtROS generation by reducing electron leakage may be one of the protective mechanisms of exercise on mitochondria.

## The impact of aging on mitochondrial gene expression and protein synthesis

Skeletal muscle mitochondrial gene expression and protein synthesis are also impacted by age. Muscle fibers of old rats (27-month) contain lower copy numbers of mtDNA than those of young rats (6-month) [19]. This was also accompanied by reduced COX transcripts in highly oxidative tissues (soleus) but not in less oxidative tissues (gastrocnemius). Likewise, in a human study, the investigators found an inverse relationship between age and the abundance of skeletal muscle mtDNA and mRNA that encoded for COX3 and COX4 [29]. Moreover, the older subjects showed higher oxidative damage (8-oxo-dG) and diminished mitochondrial ATP production rate with age.

Given an age-associated decrease in mtDNA expression, the next level of enquiry would be to examine the translational levels of mitochondrial proteins in the aged. Similar to findings in gene expression, isotopic mass spectrometry analysis of fractional rate of protein synthesis in skeletal muscle demonstrated higher (40%) mitochondrial protein synthesis rate in young humans (24 ± 1 years), compared with middle aged (54 ± 1 years) and older humans (73 ± 2 years) [20]. In this study, COX and citrate synthase enzyme activities were also inversely related to age. Thus, the implication is that genetic alterations are being carried over to the protein level and eventually manifest in changes to enzymatic activities.

The role of the mitochondria is to supply ATP to the different cells within the organism. In addition to the accrual of oxidative damage to structural components of mitochondria, functional deficits are possibly incurred, particularly in energy production. In a review of the literature by Russ and Kent-Braun [30], a common finding amongst studies was that oxidative capacity in skeletal muscle of the older adults was lower than young individuals. These studies comprised cross-sectional analyses of human subjects that differed in age and physical activity levels, and both in vitro and in vivo methods were used to measure oxidative capacity in their skeletal muscles.

## Insights on mitochondrial function from in vitro experiments

The in vitro experiments involved isolation of muscle biopsies from human subjects, followed by quantification of activity levels of oxidative enzymes such as citrate synthase, SDH and COX using fluorometric techniques. Key findings from these studies indicated that there is a decline in oxidative capacity in aged skeletal muscle as a function of age, as shown by reduced enzymatic activities. Unfortunately, the results were confounded by physical activity, which were surveyed differently in the studies. For example, some studies measured physical activity using self-reported questionnaires, while others measured whole body maximal oxygen consumption (VO_2max_) during treadmill testing [30]. The problem with this approach is that the first method does not allow the investigators to accurately assess habitual physical activity due to recall bias of the participants. Secondly, the value of VO_2max_ as a tool to assess physical activity patterns is undermined by the contribution of genetic potential—that is, some sedentary individuals are genetically endowed with high aerobic potential. Thus, it is not a true measure of physical activity habits.

These inconsistencies have prompted scholars in the field to argue that reduced oxidative capacity in skeletal muscle is probably not a correlate of age, but rather a result of declining habitual physical activity [30]. Supporting this viewpoint is the finding that no differences in citrate synthase or SDH activities existed between endurance-trained young and old individuals [30]. Therefore, it seems, reduced enzymatic activities in mitochondria of old individuals may be partly, if not mostly, attributable to decreased physical activity associated with age, rather than by the aging process itself.

An additional weakness with the in vitro assays lies in the techniques used to quantify the activities of the mitochondrial enzymes, since they give information on the function of mitochondria under an *artificial* environment: enzymatic analyses involve measuring the activities of the enzymes in solution and not in their natural environment within the mitochondria. In situ analysis of mitochondria is a solution to this problem as it allows oxidative enzymes to be studied within their normal environments, that is, inside the mitochondria. Secondly, this technique measures the integrity of the respiratory chain as well as ATP synthesis, which are more robust measures of oxidative capacity than assaying only a few enzymes of the tricarboxylic cycle. Using this technique, Rasmussen et al. [31, 32] found that out of 13 different enzyme activities assayed, age accounted for just two of the differences (β-oxidation and α-glycerophosphate dehydrogenase) in the activities, whereas the others (e.g., respiratory chain, tricarboxylic acid cycle, COX, citrate synthase, ATP synthesis) were not significantly influenced by age. In addition, State 4 respiration was not higher and P/O ratios were not lower for the older adults compared with the young subjects. Congruently, a more recent study found that while P/O ratios of older sedentary adults are markedly depressed, P/O ratios are similar between young active adults and old active adults [33], further supporting the notion that coupling efficiency is much more dependent on physical activity level than on age. Thus, the argument that reduced oxidative capacity is a consequence of aging continues to be challenged, by both results from in vitro and in vivo experiments.

## Insights on mitochondrial function from in vivo experiments

The in vivo experiments described in Russ and Kent-Braun’s review paper [30] utilize magnetic resonance spectroscopy (MRS). In general, these experiments are based on following the changes in metabolites such as ATP, PCr, Pi and changes in pH to portray real-time muscle respiration. Using MRS [34–40] to quantify muscle metabolism confers some advantages over the methodology of in vitro experiments. These advantages include: (i) obtaining a larger tissue mass than tissue biopsies that are used for in vitro assays, (ii) offering a more functional depiction of oxidative capacity in the whole muscle under physiological conditions, as opposed to isolated conditions that are reliant on non-physiological concentrations of mitochondrial substrates. Nevertheless, the in vivo experiments did not offer much resolution to the conflict over aging and mitochondrial dysfunction. Some studies showed decreased oxidative capacity in skeletal muscle from older adults, whereas others found no significant differences between young and old skeletal muscle. These studies are discussed in the following sections.

## Reduced mitochondrial coupling and sarcopenia in aging

In one study [41], VO_2_max was negatively related to age, with the older adults’ (69 ± 6 years old) oxygen consumption ~ 45% of the adult (39 ± 8 years old) value. Cross-sectional areas of the quadriceps muscles of older subjects were also 33% lower than the young adults. When corrected for smaller muscle volume in the older adults, oxidative capacity was 36% of that in the adult muscle. Another study [42], however, found no differences in oxidative capacity between the old (75 ± 5 years old) and young (33 ± 5 years old). An explanation for this discrepancy could be traced to the muscles that were studied. In the first study, the quadriceps were studied, while the tibialis anterior was analyzed for the latter. It is likely that muscle-specific differences exist in aging skeletal muscle, possibly due to the different proportions of type I and II fibers within the muscles. The tibialis anterior and the vastus lateralis are comprised of 25 and 60% type II fibers, respectively [43].

Conley’s group [43] reported no significant difference between older and younger adults for both mitochondrial coupling and ATP concentration in the tibialis anterior. On the other hand, differences in both energetic properties were apparent for the vastus lateralis, with muscle from older adults exhibiting decreased P/O and [ATP]. This disparity in mitochondrial function between type I/”slow-twitch” (aerobic/oxidative phosphorylative) and type II /”fast-twitch” (anaerobic/glycolytic) fibers supports the contributing role of ROS in mitochondrial dysfunction; type II fibers generate 2–3 times higher H_2_O_2_ than type I fibers [44]. Increased ROS production is responsible for the increased apoptotic process in type II muscle fibers, leading to preferential loss of such fibers with aging [43]. These results suggest that mitochondrial uncoupling is associated with aging in skeletal muscle and could be an underlying mechanism for inefficient energy production, lowered [ATP] within myocytes, and increased apoptosis of type II fibers that manifests as sarcopenia.

Physical activity may be a confounding factor in investigating mitochondrial dysfunction and sarcopenia, although the results from Conley et al. [43] show that the tibialis anterior, which like the vastus lateralis, is also recruited during locomotion, has comparable bioenergetic functions in older versus young adult subjects. Contrarily, differences were found for the vastus lateralis. As the subjects in this study were recreationally active, as assessed by physical activity questionnaires, the implication is that intrinsic changes within the skeletal muscle in the older population could lead to the lower energy properties, even when the older persons are physically active.

Nevertheless, the characteristic macroscopic manifestation of skeletal muscle degeneration with aging appears as follows: loss of both type I and type II fibers with preferential loss of type II fibers, as well as increase in aberrant COX^−^/SDH^++^ fibers. The physiologic mechanisms that underly and collectively engender the aforementioned phenotypic changes are both extrinsic, resulting from global and systemic changes with aging, and intrinsic, resulting from changes that occur within the myocytes and their organelles. Among the extrinsic causes are progressive denervation, altered endocrine and autocrine regulation, satellite stem cells declines, and altered protein metabolism [45, 46]. The intrinsic mechanisms, on the other hand, include the aforementioned: reduced protein synthesis, DNA damage and mtDNA deletion-mutations, as well as impaired mitochondrial dynamics and decreased autophagic and mitophagic degradation, which will be discussed in the following section. These intrinsic changes, particularly the energetic ones, are less understood and are the focus of this review.

One of the causes of fiber size and quantity decline is myofiber mitochondrial dysregulation which fosters generation of oxygen radicals which in turn damage the nuclear and mitochondrial genomes, ultimately resulting in decreased protein expression and, consequently, atrophy or even apoptosis of the myofibers. Congruent with this explanatory model, it has been demonstrated that mitochondrial free radical generation (H_2_O_2_ produced/O_2_ consumed) is two- to threefold higher in type IIB fibers than in type I fibers in situ [47]*.* This intrinsic difference in myofiber properties most likely accounts for the accelerated decline of type II fibers, particularly type IIB, with aging.

### Compromised mitochondrial quality control and impaired mitophagy with aging

A defining element of mitochondrial dynamics and a critical one to upholding mitochondrial integrity is constant turnover via cycles of generation, dynamics and clearance of the mitochondrial reticulum achieved by the processes of biogenesis, fusion/fission, and mitophagy respectively. Of these, biogenesis is the most well studied. The process of mitochondrial biogenesis, the expansion of the existing mitochondrial network through both growth and division, is constantly ongoing in myofibers but declines with aging [48]. However, aged skeletal muscle possesses a reduced capacity to induce biogenesis in response to biogenesis-inducing stimuli such as muscular contraction [49]. Peroxisome proliferator-activated receptor gamma coactivator 1α (PGC-1α) is the master regulator of mitochondrial biogenesis, along with other purposes such as glucose and fatty acid metabolism [50]. PGC-1α serves as a coactivator for a number of nuclear genes encoding mitochondrial proteins, one of which is transcription factor A of the mitochondria (Tfam), a critical regulator of mitochondrial biogenesis and coordinator of nuclear and mitochondrial genomes [51]. PGC-1α, along with Tfam, has been found to decrease with age in rodents, but aerobic exercise has been well demonstrated to attenuate these declines and increase PGC-1α, Tfam, and nuclear respiratory factor 1 (NRF1), the three key inducers of mitochondrial biogenesis. For example, just 12 weeks of aerobic exercise in older rats attenuated age-related declines of PGC-1α and Tfam, restoring expression to levels even higher than that of young untrained rats [52]. Likewise, aerobic training in both older and younger adults has been demonstrated to increase PGC-1α expression by 55% [53]. Although human results have been inconsistent, age-related reductions in PGC-1α may be partly responsible for the decline in mitochondrial volume in aged skeletal muscle. Thus, upregulating PGC-1α and TFAM, and thereby augmenting mitochondrial biogenesis, may be one avenue by which exercise preserves muscle mitochondrial quality and myofiber quality.

Mitochondrial quality is tightly regulated via fine balance between fusion and fission, two opposing processes which are constantly reshaping mitochondrial architecture to optimize for functional demands. Fusion refers to the spatial expansion of the mitochondrial network primarily via proteins mitofusin 1 (Mfn1) and mitofusin 2 (Mfn2). In contrast, mitochrondrial fission is the process of dividing the network into fragmented, globular mitochondria. The balance between these two processes is critical to upkeeping effective mitochondria and skeletal muscle function, but with aging is often compromised. Some studies indicate that aging confers dysregulation in the direction of disproportionately elevated fission [54], consistent with the fragmented mitochondrial structures often observed in aged rodent skeletal muscle [55]. Particularly, Mfn2 levels appear to decline progressively with age as demonstrated in rodent studies, a deficiency that drives mitochondrial dysfunction and impairs mitophagy [56]. Conversely, aerobic training in older adults increases the ratio of fusion to fission proteins, favoring a more fused, tubular mitochondrial network [57]. Congruently, another key operator in fusion, mitochondrial protein, optic atrophy 1 (OPA1), in addition to Mfn1, has been shown to decline with age in sedentary, but not active, adults [58]. Age-related decline of skeletal muscle Opa1 is likewise apparent in mice, but, in one study, only 1 week of aerobic exercise proved sufficient to reactivate its expression in aged mice [58]. Therefore, aerobic exercise appears to be a putative intervention to rebalance the scale of mitochondrial dynamics by upregulating mitochondrial fusion.

One possible fate of mitochondrial fragments post-fission is lysosomal degradation via a process known as mitophagy, mitochondria-targeted autophagy. Mitophagy is critical in clearing aberrant or diseased mitochondria. However, with age, mitophagy declines [59], particularly in skeletal muscle [60]. Compromised mitophagy predictably results in mitochondrial damage accumulation, augmented oxidative stress, and increased apoptosis, which together culminate in both myopathy and muscular atrophy [61]. Thus, impaired mitophagy is likely a key player in the pathophysiological progression of sarcopenia and is a potential target for intervention.

Although understanding of its role is only recently emerging, mitophagy increasingly appears to be a critical mechanism of exercise-induced remodeling, but human data on the matter remain imperfect [62, 63], as studies have relied upon indirect indicators of mitophagy: changes in gene expression or protein concentration of mitophagic regulators. While the complexities of both mitophagy and the innovative methods of its detection are beyond the scope of the review, what is of pertinence is that mitophagy may be one of the key avenues by which exercise rescues myofiber mitochondria. In one rodent study, for example, aerobic exercise resulted in enhance mitophagy flux, as well as enhanced targeting of mitochondria for degradation, increasing mitochondrial turnover; moreover, this outcome appeared to be PGC-1α dependent, as results were not replicated in PGC-1α^−/−^ mice [64]. Remarkably, another study in mice demonstrated that a single bout of treadmill running stimulates post-exercise autophagy 3 times higher than that of basal expression levels in skeletal muscle, in an AMPK/Ulk1-dependent manner [65]. Like aerobic training, resistance training also appears to be a potent inducer of skeletal muscle autophagy. For example, in one study, the skeletal muscle of 18–20 month old rats that underwent 9 weeks of resistance training exhibited enhanced autophagy and reduced myocytic apoptosis, presumably via inhibition of the Akt/mTOR pathway and activation of the FOXO3a pathway [66].

## Exercise training in older adults—can it restore mitochondrial energetics?

### Endurance training

Although the exact nature of mitochondrial dysfunction in aging has yet to be determined, there is consensus that regular exercise can help maintain muscle mass and aerobic fitness [67]. Endurance training (ET) is well known to improve oxidative capacity [68] and induce mitochondrial biogenesis [69] in skeletal muscle. ET acts multifariously to improve muscle quality and energetic coupling in older individuals and thereby holds great potential in ameliorating sarcopenia. ET has been well documented to prevent age-associated loss of oxidative capacity [70]. For example, in one study, endurance-trained older men (51–62 years old) possessed SDH activities in type I and IIa muscle fibers at levels comparable to younger (21–30 years old) endurance-trained individuals. This elevation in enzyme activity persisted even though the older trained individuals, like their untrained, age-matched counterparts, had smaller cross-sectional areas of type II fibers than the younger men. This study encompassed a cross-sectional design, with subjects recruited based on their habitual physical activity. Hence, it is not clear as to the temporal sequence of whether regular exercise maintains muscle oxidative capacity or vice versa. More recently, another study assayed vastus lateralis biopsies of young and old endurance-trained men vs. age-matched sedentary men. Oxidative phosphorylation proteins were universally elevated in the active groups, and COX activity significantly higher in active old individuals than in their sedentary counterparts, supporting the findings of the aforementioned study [71].

However, numerous interventional trials have since elucidated the temporal sequence, confirming the fact that ET improves muscle oxidative capacity. While the aforementioned findings lack causative rigor, ET, when performed in clinical interventions, consistently improves muscle health metrics and energetics, especially in aged individuals. For instance, increases in mtDNA and activity of the ETC, particularly of complex I and complex II, were observed after 12 weeks of ET in healthy older (67.3 ± 0.6 years old) persons that were previously sedentary [28]. The observed improvements in mitochondrial function were thought to be a result of observed increases in mitochondrial biogenesis. Congruently, Short and colleagues [14] reported that 16 weeks of ET in men and women aged between 21 and 87 years conferred not only improvements in peak oxygen uptake (VO_2_peak), but also increases in skeletal muscle mitochondrial enzyme activities and mRNA expressions (citrate synthase and COX) and genes involved in mitochondrial biogenesis (PGC-1α, NRF-1, TFAM). These changes occurred regardless of age, indicating that biological adaptations to exercise training were preserved in aged skeletal muscle. A caveat in this study lies in the small sample size for each age-group, with approximately 6 subjects per decade. This could potentially limit the generalizability of the results. Furthermore, another limitation of the study is that protein synthesis was not measured for the genes that mediate mitochondrial biogenesis; thus, whether exercise-induced changes in gene expression was translated to the protein level was not confirmed.

Perhaps the most comprehensive study on the potential of ET to rescue age-related mitochondrial energy decline is the work of Broskey et al. [27]. First, they found that active individuals exhibited not only an average mitochondrial volume density 48.9% greater than that of age-matched sedentary individuals, but also significantly higher activity of electron transport complexes I, IV, and V. Second, they found that when sedentary individuals engaged in moderate-intensity endurance exercise for 16 weeks (diet remaining unchanged), not only did mitochondrial volume density increase by 50.7% concomitant with increases in PGC-1α and TFAM gene expression, but electron transport complexes III, IV, and V were also dramatically upregulated. These findings together confirm that physical activity level is a greater determinant of mitochondrial energetic capacity than aging itself, and thus the observed mitochondrial decline in aged individuals is likely more so an outcome of decreased activity levels, rather than of aging itself. A caveat of the implications of the study lies in the fact that while oxidative capacity was indeed increased by aerobic exercise, the actual ratio of ATP_max_ to mitochondrial volume density remained unchanged. That is, oxidative capacity improved not due to functional changes in oxidative coupling efficiency, but instead due to increased mitochondrial biogenesis and thus increased mitochondrial volume.

While the previously cited trials indicated that oxidative capacity improves secondary to augmented mitochondrial biogenesis, other studies have reported improvements in phosphorylation capacity and ATP_max_ independent of or disproportionate to changes in mitochondrial volume [72, 73], suggesting intrinsic changes in mitochondrial function. Conley et al. [72] found that 6 months of ET of aged individuals (69.5 ± 1.2 years old) resulted in a significant increase in ATP_max_ as well as a 31% increase in oxidative capacity, but was unaccompanied by any increase in mitochondrial volume, indicating improved coupling efficiency [72]. That is, the ratio of phosphorylation capacity to the ratio of mitochondrial volume (ATP_max_/V_V_) increased, hence intrinsic energy coupling increased. An important difference between this and aforementioned ET studies may account for these disparate findings. That is, the training modalities and intensities in this study differ from other ET protocols: while most of the aforementioned studies involve biking, running, rowing, or walking at a moderate intensity (~ 75% of maximum heart rate), in this study, subjects used a “one-legged press” StairMaster machine as well as a kayak-simulating machine at a heart rate of 80–85% [73]. Both of these, in addition to being conditioning exercises, are weight-loaded and therefore possess some element of resistance training, as reflected in the greater weight lifted post-intervention and the relatively small increase in VO_2_max (only 5.4%) compared to that of the other ET studies, despite the greater programme length. Thus, the question is raised: do the mitochondrial benefits conferred by exercise differ based on training modality—increased mitochondrial volume for ET and enhanced coupling efficiency for resistance training? This will be investigated in the following section.

Regarding ET intensity, it seems that higher intensity training confers more rapid, more significant improvements in mitochondrial respiratory capacity. In a study by Granata et al. [74], 4 weeks of sprint-interval training (4–10 × 30 s all-out bouts at ~ 200% of peak power output), but not HIIT, or sublactate threshold continuous training, increased maximal mitochondrial respiration as well as PGC-1α and p53 protein content, as well as mitochondrial respiration, but not mitochondrial content or ETC subunit content. Congruently, another study found that a single bout of high intensity (80% of VO_2_ max) cycling engendered a 10.2-fold increase in PGC-1α mRNA while isocaloric low intensity (40% of VO_2_ max) engendered an only 3.8-fold increase PGC-1α mRNA [75]. A worthwhile consideration for this manifestation is differential fiber recruitment and differential fiber response properties. Specifically, training at 80% of VO_2_ max recruits primarily type II fibers while training at 40% of VO_2_ max recruits primarily type I fibers. Work by Russell et al. [76] has indicated that type II fibers possess a greater capacity to upregulate PGC-1α transcription in response to training stimulus. Specifically, after 6 weeks of interval training at 70–80% of VO_2_ max, an intensity similar to the high intensity group in the aforementioned trial, PGC-1α protein content increased 2.8-fold in type IIa fibers and 1.5-fold in type I and type IIx fibers. Thus, the greater induction of PGC-1α expression via high intensity ET than moderate intensity ET is likely attributable to its greater recruitment of type II fibers. Interestingly, a recent study found that continuous endurance training (~ 65% of VO_2_ max) and HIIT (~ 170% of VO_2_ max) for 6 weeks confer very similar changes in intramuscular signaling and even fiber-type recruitment, with almost no differences in outcome with the exception being that HIIT training conferred greater improvements in anaerobic and glycolytic capacity [77].

In addition to its energetic impacts, ET also modulates the level of oxidative damage in skeletal muscle. Two animal studies [78, 79] demonstrated that treadmill running for 8 or 10 weeks attenuated the content of oxo8dG and increased the activities of Oxoguanine DNA glycosylase (OGG1) and Uracil DNA glycosylase (UDG), respectively, in skeletal muscle of aged (30-month and 21-month-old) rats. It is inferred that at least in animal studies, exercise exerts a positive effect on aged skeletal muscle by reducing oxidative damage through upregulation of DNA repair mechanisms. In addition to upregulating DNA repair mechanisms and bolstering antioxidant defense mechanisms [80], ET may also reduce oxygen damage by mitigating electron leakage, by improving electron flux, and thereby reducing ROS generation.

Another potential benefit of endurance training, as mentioned in earlier sections, is its ability to reverse age-associated declines in autophagy in skeletal muscle. After 8 weeks of treadmill running 5 × per week, key autophagic regulators autophagy-related gene 7 and beclin-1 expression levels were restored in 12 month old mice [81]. Such findings are consistent with other studies and similar upregulations in autophagy can be seen even with single bouts of training. For example, in one study [82], following a single bout of swimming, several autophagic proteins, Atg5, Atg7, p62, and LC3-II, were upregulated and Ulk1 phosphorylation, a key state in autophagic regulation, was increased in the gastrocnemius of 24 month old mice. Moreover, the effect of aerobic training on skeletal muscle autophagy may be dependent on exercise intensity. In one study, moderate intensity treadmill running (20 m/min) predictably increased autophagy gene expression. Conversely, however, high intensity (30 m/min) treadmill running, which is above the mouse lactate threshold of 20 m/min, resulted in diminished autophagy gene expression (83). Low intensity running, however, engendered no significant changes in autophagy gene expression. Thus, aerobic exercise seems to induce autophagy in a bimodal response and may be optimized with moderate intensity ET.

### Resistance training

Muscle strength declines with aging to a greater degree and at a greater rate than does muscle mass, indicating a decrease in muscle “quality” with age [84]. Skeletal muscle loss with aging is primarily attributed to a loss of type II muscle fibers [85], which is closely related to muscle “quality” and peak force which diminishes with sarcopenia. Resistance training (RT) directly increases type II muscle fiber size and number [85] and therefore is a worthy candidate for intervention against sarcopenia. While the evidence for the mitochondrial benefits of ET are robust, the effects of RT on mitochondrial coupling has been much less studied. All resistance training is not created equal—the repetition range is highly determinate of which energy systems are utilized and therefore of how mitochondria are recruited and modulated. Thus, as most RT can be classified as anaerobic activity, with the exception of high repetition training, RT, in theory, has less to do with mitochondria than does ET. So while the aforementioned benefits of RT in increasing muscle mass and strength, maintaining function and mobility, and improving metabolic health have been widely demonstrated [86], RT’s mitochondrial effects are less established. Nevertheless, recent evidence indicates that RT may also improve skeletal muscle mitochondrial function and restore muscle energetics in older individuals, with early evidence in older adults demonstrating that chronic RT intervention not only phenotypically ameliorates muscle weakness, but also markedly reverses the transcriptional signatures of age-associated mitochondrial impairment, restoring the mitochondrial transcriptome to that of a younger adult [87].

Skeletal muscle hypertrophy is typically accompanied by new myonuclei within muscle fibers, which are primarily derived from myosatellite cells, or muscle stem cells. However, recent work indicates a differential relationship between myonuclear accrual and muscular hypertrophy between type I and type II fibers, suggesting differential mechanisms of hypertrophy between the two types. Following 12 weeks weeks of resistant training in older (71 ± 4.4 years old) adults, type II fiber hypertrophy was predictably much greater than type I fiber hypertrophy (23 vs 8%), despite myonuclear content only increased in type I fibers [88]. The authors, Moro et al. [88], thus speculate that type II myonuclei are able to upregulate transcriptional activity to a degree that supports RT-induced hypertrophy in the absence of satellite cell differentiation or myonuclear accrual. This reflects one of a number of ways in which hypertrophic processes differ by fiber type.

Porter et al. [89] found that 12-week full-body RT at a range of 3–4 sets of 8–10 repetitions increased electron transfer capacity by 65%, and significantly enhanced maximal coupling respiration along with intrinsic mitochondrial function, without augmenting mitochondrial biogenesis or volume. Interestingly, while coupled respiration of NADH via complex I was significantly increased by RT, complex II activity only slightly increased. That is, RT increased both complex I and complex II activity as it increased total mitochondrial respiratory capacity, but appreciably shifted the relative contribution towards complex I. Moreover, the mRNA of COX4I1 (Complex IV) and NAMPT, which controls synthesis of NAD^+^, was elevated. Unfortunately, this study is limited in scope as its subjects were all young; nevertheless, it shows that RT indeed enhances oxidative coupling in skeletal muscle, and that it does so independently of mitochondrial biogenesis or any increase in mitochondrial volume, consistent with our previous speculation on RT.

Similar improvements have been demonstrated to be conferred by RT in aged individuals. In a study by Parise et al. [2], 28 older adults (age 68.5 ± 5.1 years) performed whole-body RT three times per week for 14 weeks ranging between 10 and 12 repetitions per set for 3 sets with 2 min of rest between each set [2]. Following this RT intervention, ETC complex IV activity increased along with mitochondrial creatine kinase content, while mitochondrial mass, mtDNA deletions, and other ETC complex activities remained unchanged [2], suggesting again a qualitative improvement in mitochondrial function independent of mitochondrial volume. More interestingly, RT reduced oxidative stress as measured by a 17.5% decrease in urinary 8-OHdG, without any change in antioxidant enzyme protein content [2]. Thus, it seems that RT may decrease oxidative stress not by inducing antioxidant activity, but rather by reducing ETC electron leakage by enhancing ETC electron flux, as observed in the increase in complex IV/I + III ratio [2]. However, any potential antioxidant benefits of RT must be taken with a grain of salt as a recent 2020 meta-analysis including 614 old (average age 68.1) individuals concluded that RT is not effective in reducing global markers of oxidative stress [90]. Nevertheless, it could be possible that RT, by reducing electron leakage, still reduces oxidative stress locally within skeletal muscle mitochondria without altering global markers of redox homeostasis.

Likewise, a recent 2020 study [91] reported significant increases in the abundance of oxidative phosphorylation complex proteins I–V without any significant change in mitochondrial biogenesis proteins, PGC-1α, TFAM, and NRF1, following 10 weeks of full-body RT in untrained old (59 ± 4 years) adults, further confirming the idea that RT acts to enhance qualitative mitochondrial capacity by improving coupling efficiency. Additionally, mitochondrial fusion proteins Mfn1, Mfn2, and Opa1 were found to be elevated. A previously mentioned trial by Jubrias et al. [73] featured not only the aforementioned ET group, but also an RT group. The untrained older individuals (average age 69.2 ± 0.6 years) participated in a periodized RT program consisting of both lighter days (3 sets of 10–15 repetitions) and heavy days (3–5 sets of 4–8 repetitions) for a total of 3 sessions per week over a 24-week period. Remarkably, this group experienced an increase in skeletal muscle oxidative capacity even greater than that of the ET group. However, this 57% increase in oxidative capacity was accompanied by a 31% in mitochondrial density in addition to 10% increase in skeletal muscle mass, as expected. While these results do show that RT can, in some cases, induce mitochondrial biogenesis, it is nevertheless consistent with the idea that RT increases the ratio of oxidative capacity to mitochondrial volume, therefore improving coupling efficiency.

In contrast, low volume and low repetition RT appear to be ineffective at inducing mitochondrial changes, despite improving muscular strength. For instance, a study by Flack et al. [92] reported unchanged oxidative capacity and ROS production following 12 weeks of full-body RT 3x/week in untrained older (≥ 60-year-old) males. One factor in this study that sets it apart from the other RT studies, and is likely responsible for its disparate outcomes, is that subjects performed only one set of each exercise per session. Thus, low volume RT appears inept at inducing mitochondrial changes.

Like low volume RT, low repetition RT, or maximal voluntary contraction (MVC) RT seems not to carry the same mitochondrial benefits as moderate-to-high repetition, and volume training. Demonstrating this, a recent study found that when 10 older adults (75 ± 9 years) underwent an 8-week regiment of MVC knee-extension quadriceps training, maximal ADP-stimulated respiration rate actually decreased in conjunction with decreased complex I activity and peak oxidative ATP synthesis [93]. One caveat of this study is that, as opposed to other RT studies involving full-body training, this study only involved performing one isolation exercise- single leg knee extensions. Nevertheless, these findings must be considered in light of the functional skeletal muscle improvements conferred by resistance exercise, as illustrated in multiple interventional studies demonstrating its ability to ameliorate severe sarcopenia [94] and protect against progression in pre-sarcopenia [95]. Moreover, these results make sense in light of the fact that low repetition MVC training as executed in the study, is entirely anaerobic, deriving most of its energy glycolytically as well as from phosphocreatine and therefore has little to do with mitochondrial processes or type I oxidative fibers.

Thus, as the repetition range increases (and the %MVC decreases), the benefits of RT become increasingly oxidative and energetic in nature, overlapping to a greater degree with the improvements conferred by ET. So while low repetition MVC RT confers functional benefits that attenuate sarcopenic dysfunction, such improvements may come completely independent of mitochondrial changes. An additional advantage of high repetition RT is that it may be more accessible to older individuals who lack the endurance necessary to engage in ET forms such as running or cycling or may risk injury bearing heavy loads with higher % MVC training. Moderate repetition RT, on the other hand, improves oxidative capacity as does ET, and even appears to be more effective at improving mitochondrial coupling efficiency and inducing qualitative changes than ET. Given that ET, such as running, may be unfeasible for older individuals, we speculate whether high [15–20] repetition RT would garner similar mitochondrial improvements to ET or CT and be a safer and more accessible option for older individuals. While it has been demonstrated that high repetition, low load RT can be equally as effective as low repetition, high load RT in inducing muscle hypertrophy [96, 97], the effects of high repetition training on mitochondrial energetics remain unstudied, and thus warrant investigation.

### Concurrent training—the best of both worlds?

Given the independent benefits of endurance and resistance training on mitochondrial aging and sarcopenia, the potential of combined endurance and resistance exercise is worthy of investigation. RT and ET each hold their own unique benefits in the context of sarcopenia and aging. RT serves to maintain and improve mobility, strength, and movement in a way that both protects against joint degeneration and preserves skeletal muscle function. ET improves mitochondrial energetics, thereby enhancing metabolic flexibility as well as increasing “muscle quality”. These are highly synergistic improvements, working together to stave off both the molecular and macroscopic changes that are incurred with aging. Therefore, concurrent, or “combined”, training is a putative intervention to preserve energetic and functional health of skeletal muscle with aging.

One study demonstrated that RT actually amplifies induction of mitochondrial biogenesis by ET when performed subsequent to ET, as seen in significantly higher increases in PGC-1α and PRC [98]; In addition, RT and ET together, but not ET alone, effectively activated mTOR signaling [98]. Concurrent training (CT) may alter adaptations compared to single mode training, but the mechanisms of interaction and the implications of so-called “interference” caused by antagonistic signaling are poorly understood.

In a comprehensive clinical trial by Irving et al. [99], both old and young sedentary individuals underwent 8 weeks of either ET, RT, or CT. While all three training modalities predictably resulted in positive changes in muscle strength and cardiorespiratory fitness, only ET and CT engendered beneficial mitochondrial changes and improvements in oxidative phosphorylation capacity. Interestingly, CT produced even more robust energetic benefits than ET alone despite consisting of 50% of the aerobic volume of the solely ET protocol (30 min vs 1 h of cycling, respectively), suggesting that RT somehow amplifies the beneficial effects of ET when performed adjunctly. CT moreover appeared to be the most effective in improving overall muscle quality in the older subjects. Also of note is that the RT and CT groups performed 4 sets of 8–10 repetitions, a much higher, and therefore somewhat more aerobic, repetition range than that in the aforementioned low repetition, MVC RT study, which reported no improvements in oxidative capacity. While this study does not specify whether CT was performed in the order of RT followed by ET, or vice versa, other data has suggested that order of CT is mostly inconsequential to mitochondrial adaptations [100].

Specifically, this study [100] reported similar improvements following 6 weeks of CT, albeit in young adults, and found that training sequence had little impact on outcome. Prior to intervention, young adult subjects who were previously sedentary for 6 months completed 2 weeks of knee joint immobilization to induce muscle atrophy and disuse. Once the brace was removed, subjects engaged in a session of either RT followed immediately by ET, or strength-endurance (SE) training, or ET followed immediately by RT, or endurance-strength (ES) training. After this acute bout of exercise, mitochondrial biogenesis was induced with PGC-1α increased tenfold and PRC increased by 570% with little difference between the groups of differing protocol order [100]. Then, over the next 6 weeks participants performed SE or ES and were assayed for mitochondrial protein expression. CT increased protein content for all five ETC complexes as well as citrate synthase and COX. Importantly, ordering of training modality had negligible influence on molecular or performance effects, with one exception being that complex II saw increased gains in the SE group than the ES group [100]. While some studies indicate that CT order does not affect strength adaptions [101], other studies [102] as well as a recent meta-analysis [103] of such studies have concluded that SE is superior to ES in that performing RT first confers greater strength gains, without affecting aerobic adaptations. Moreover, a 2020 study found that while SE and ES training equally induce mitochondrial biogenesis, SE training additionally activates mTOR signaling [104]. Thus, it is a safe bet to sequence RT before ET in anti-aging exercise protocols (Table 1).

**Table 1:** Oxidative mitochondrial outcomes of RT,  ET and CT  interventions

Authors	Cohort	Training type	Intensity [ET]/repetition range [RT]	Intervention duration/frequency	Primary outcomes
Menshikova et al. [28]	Sedentary older (67 ± 0.6 years) adults (5 men, 3 women)	ET	30–40 min of HR 50–70% of VO_2_ max (progressive)	12 weeks 4-6x/week	↑ETC Activity ↑NADH Oxidase (CI) ↑mtDNA/mitochondrial biogenesis ↑Complex I-IV activity
Short et al. [14]	Untrained men/women (21–87 years)	ET	20 min at 70% of max HR (start) 40 min at 80% of max HR	16 weeks 3x/week (start) 4x/week (end)	↑Complex IV activity ↑Citrate synthase ↑COX4, ND4 (mito. Enzymes) ↑PGC-1α, TFAM, NRF-1
Broskey et al. [27]	Sedentary men and women (60- 80 years)	ET	75% of max HR for 30–60 min (progressive along course of intervention)	16 weeks 3x/week	↑ETC Complexes I, III, IV, V w/ greatest elevation of I ↑Mitochondrial volume (proportional to ↑ATPmax) ↑PGC-1α, TFAM, NRF-1
Conley et al. [72]	Recreationally active older (69.5 ± 1.2 years) men and women	ET	60%—> 80–85% of max HR for 20 min on two different exercises	24 weeks 3x/week	↑ATPmax (+ 31% oxidative capacity) (Disproportionate to change in Mito vol.)
Jubrias et al. [73]	Untrained older (69.2 ± 0.6 years) men and women	RT (Leg press + arm training + shoulder lifts)	3 sets of 10–15 repetitions (lighter days) 3–5 sets of 4–8 repetitions (heavier days)	24 weeks 3x/week	↑57% skeletal muscle oxidative capacity (Greater than that of corresponding ET group) ↑31% mitochondrial density
Mesquita et al. [91]	Untrained aged (59 ± 4 years) men and women	RT (Full Body)	3 sets of 10–12 repetitions with 1 min rest between sets	10 weeks 2x/week	↑OXPHOS Proteins: ↑180% CI, 39% CII, 89% CIII, 43% CIV, 78% CV -Unchanged PGC-1α and TFAM
Porter et al. [89]	Untrained young men	RT (Full-body)	3–4 sets of 8–10 repetitions with 1–2 min rest between sets	12 weeks 3x/week	↑↑ETC Complex I activity ↑Coupled (P) and uncoupled mitochondrial respiration ↑COX4I1/NAMPT mRNA
Flack et al. [92]	Untrained older (≥ 60 years) males	RT (Full-body)	1 set of 8–12 repetitions	12 weeks 3x/week	No change in oxidative capacity or ROS production
Parise et al. [2]	Untrained older men/women (68.5 ± 5.1 years)	RT (Full-body)	3 sets of 10–12 repetitions with 2 min rest between sets	14 weeks 3x/week	↑ETC Complex IV activity ↓Oxidative stress ↓17.5% 8-OHdG
Berg et al. [93]	Older sedentary adults (7 men, 3 women, age 75 ± 9 years)	RT (Single muscle group MST)	4 sets of 4–5 repetitions with 2 min rest between sets	8 weeks 3x/week	↓Complex I Activity ↓Maximal ADP-dependent respiration
Irving et al. [99]	Older (≥ 61 years) and young (18–30 years) sedentary men and women	RT	4 sets of 8–10 repetitions	8 weeks 4 days/week	*-No significant change in oxidative capacity* ↑PGC1α1.SIRT3,
		ET	65% VO_2_ max for 1 h	8 weeks 5 days/week	*↑*OXPHOS ETC protein abundance ↑Oxidative Capacity ↑Complex I/Complex II ↑PGC1α1, SIRT3
		CT (ET + RT)	65% VO_2_ max for 30 min, 5 days/week + 2/3 of RT group’s resistance volume, 4 days/week	8 weeks	*↑↑*OXPHOS ETC protein abundance ↑↑Oxidative capacity ↑TFAM (mitochondrial biogenesis)
MacNeil et al. [100]	Sedentary (for 6 months) young adults	CT (RT- > ET or ET- > RT)	65% VO_2_ max for 22.5 min, immediately followed by 3 sets of 10 repetitions with 1 min. rest, and vice versa	6 weeks 3x/week	↑↑ETC complex proteins I–V ↑↑Mitochondrial biogenesis (PGC-1α, PRC) Sequence of RT > ET is superior than ET > RT at upregulating complex II

## Conclusions and future directions—how can we stave off sarcopenia and restore muscle mitochondrial health?

Mitochondrial dysfunction in aged skeletal muscle is attributable to both the aging process, as well as to a reduction in physical activity. Although regular exercise may not abolish dysfunction completely, it can help to prolong specific functional aspects of the skeletal muscle and increase healthspan. This review has described the mechanisms involved in the deterioration of muscle function with age and has detailed how exercise training intercepts these processes.

**Fig. 1:**
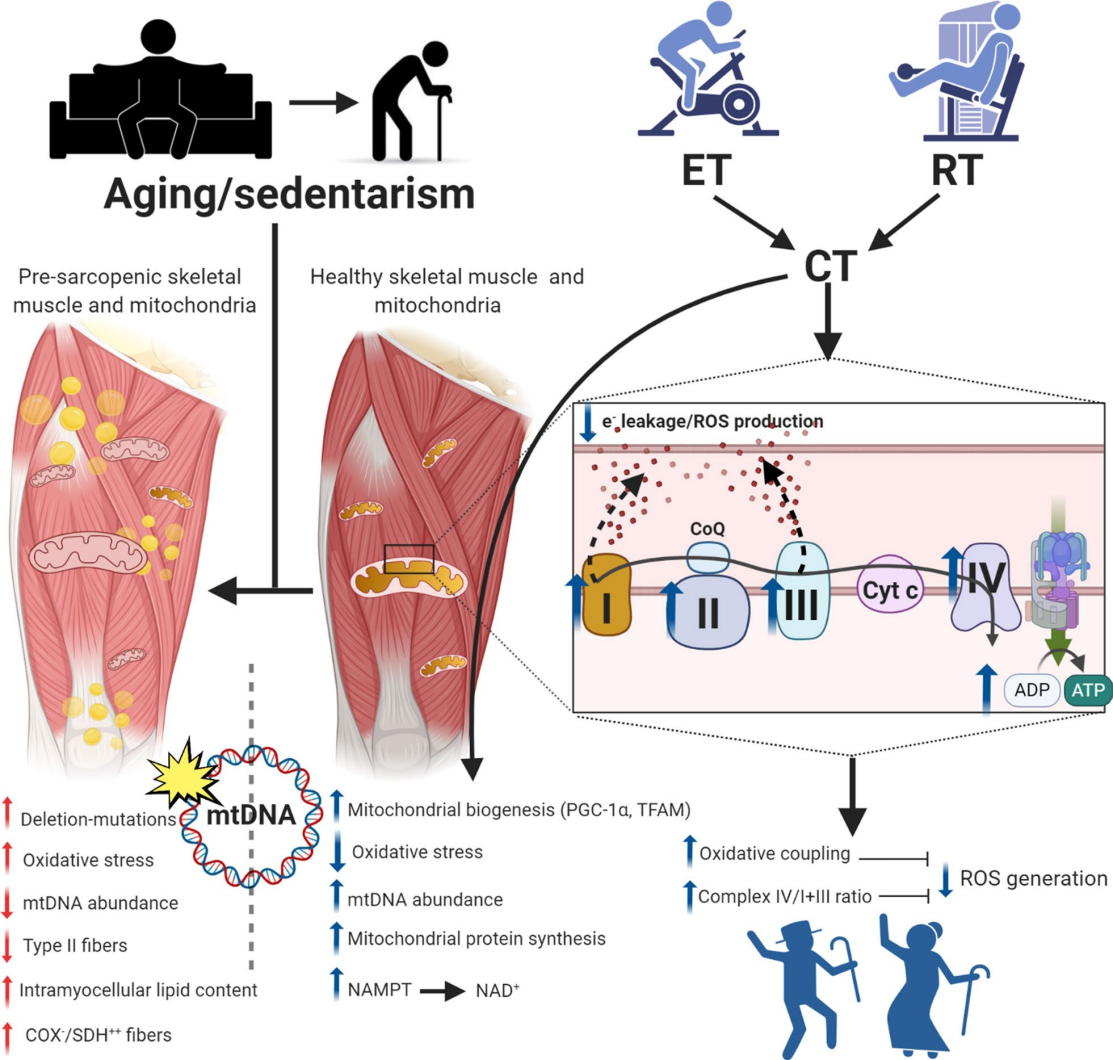
Concurrent training as a mitoprotective and anti-sarcopenic intervention. Exercise, both in the forms of endurance and resistance training, improves ETC electron flux and mitochondrial oxidative coupling. Specifically, resistance training increases the ratio of complex IV/complex I + III, which in turn minimizes electron leakage and thus ROS generation from complexes I and III, the main cellular sources of superoxide. At the level of mtDNA, this decreases oxidative damage of mtDNA and reduces prevalence of deletion-mutations. In addition, when RT is coupled with endurance training, or with endurance training alone, mtDNA abundance, mitochondrial protein synthesis, and mitochondrial biogenesis all increase. At the level of muscle fibers, these exercise-induced changes prevent age-associated aberrant COX^−^/SDH^++^ phenotypes and preserve type II muscle fibers, altogether functionally staving off sarcopenia. Figure created with BioRender.com

We propose that moderate-to-high repetition RT followed by rotating high and moderate intensity ET is a putative strategy to reverse the molecular signatures of skeletal muscle aging, restore respiratory coupling efficiency and thereby reduce chronic ROS generation/mtDNA damage, and reverse the mitochondrial transcriptome toward that of a younger adult. On a phenotypic level, this effectively staves off sarcopenia, preserving muscle function and strength in addition to improving metabolic health (Fig. 1).

## Data Availability

Not applicable.

## Abbreviations

ATP: Adenosine triphosphate
mtDNA: Mitochondrial DNA
NADH: Nicotinamide adenine dinucleotide
FADH_2_: Flavin adenine dinucleotide
ETC: Electron transport chain
ADP: Adenosine diphosphate
ROS: Reactive oxygen species
P/O ratio: ATP produced per O_2_ consumed
PCR: Polymerase chain reaction
COX: Cytochrome-c oxidase
COX^−^/SDH^++^: Cytochrome-c oxidase negative/succinate dehydrogenase-hyperreactive
MRS: Magnetic resonance spectroscopy
ET: Endurance training
UDG: Uracil DNA glycosylase
OGG1: Oxoguanine DNA glycosylase
RT: Resistance training
COX4I1: Complex IV
NAMPT: Nicotinamide phosphoribosyltransferase
MVC: Maximum voluntary contraction
CT: Concurrent training
SE: Strength-endurance
ES: Endurance-strength
